# Chemometric Analysis of Fatty Acids Profile of Ripening Chesses

**DOI:** 10.3390/molecules25081814

**Published:** 2020-04-15

**Authors:** Agnieszka Białek, Małgorzata Białek, Tomasz Lepionka, Małgorzata Czerwonka, Marian Czauderna

**Affiliations:** 1 Department of Animal Improvement and Nutrigenomics, Institute of Genetics and Animal Breeding, Polish Academy of Sciences, Postępu 36A Jastrzębiec, 05-552 Magdalenka, Poland; 2 The Kielanowski Institute of Animal Physiology and Nutrition, Polish Academy of Sciences, Instytucka 3, 05-110 Jabłonna, Poland; mr.czauderna@gmail.com; 3 Laboratory of Hygiene, Food and Nutrition, Military Institute of Hygiene and Epidemiology, Kozielska 4, 01-163 Warsaw, Poland; tomasz.lepionka@wihe.pl; 4 Department of Bromatology, Medical University of Warsaw, Banacha 1, 02-097 Warsaw, Poland; malgorzata.czerwonka@wum.edu.pl

**Keywords:** ripening cheese, fatty acids, conjugated fatty acids, odd and branched fatty acids, GC-MS, Ag^+^-HPLC-DAD, cluster analysis, principal component analysis, linear discrimination analysis, chemometrics

## Abstract

The number of different types of cheese worldwide exceeds 4000 and dairy fat, composed of about 400 fatty acids (FA), is one of the most complex dietary fats. Cheeses are valuable sources of different bioactive FA, i.e., conjugated FA (CFA). The aim of present study was to determine FA profile of commercially available ripening cheeses, with the special emphasis on CFA profile. Multivariate analyses (cluster analysis (CA), principal component Analysis (PCA), and linear discriminant analysis (LDA)) of chromatographic data have been proposed as an objective approach for evaluation and data interpretation. CA enabled the differentiation of ripening cheeses from fresh cheeses and processed cheeses. PCA allowed to differentiate some types of ripening cheese whereas proposed LDA model, based on 22 analyzed FA, enabled assessing cheeses type with average predictive sensitivities of 86.5%. Results of present study clearly demonstrated that FA and CFA content may not only contribute to overall nutritional characteristics of cheese but also, when coupled with chemometric techniques, may be used as chemical biomarkers for assessing the origin and/or the type of ripening cheeses and the confirmation of their authenticity, which is of utmost importance for consumers.

## 1. Introduction

The name ‘cheese’ is the generic name for a group of fermented milk-based food products, produced in a great range of flavors and forms throughout the world [1]. As one of the most ancient forms of manufactured food cheese has a very long history, almost as long as a mankind. Cheese-making could go as far back as 10,000 BC when sheep and goats were first domesticated in the Middle East [2]. Cheese is defined as the fresh or matured product obtained from the coagulation of milk [3]. Although cheese manufacture is practised world-wide, primarily it is a product of European countries and those populated by European emigrants. In Asia, Africa, and Latin America where tradition of cheese manufacturing and the number of cheese types is much smaller, still there are other types of dairy products (e.g., yogurt drinks in Arabic countries) considered as traditional food [1]. Technological progress has led to a multitude of different types of cheese on the market, varying in texture and flavor [2]. The majority of cheeses are produced by rennet coagulation, however in few exceptions, a plant proteinase from the *Cynara cardunculus* (e.g., Serra de Estrela (Portugal), acid (aspartate), or proteinases of animal or fungal origin are used [1]. Curd cheese (CC) is an example of fresh cheeses obtained simply by acidification of milk and thus, its FA profile is the most similar to FA profile of raw milk used for production. As the opposite, a processed cheeses (PC) can be considered. They are produced by grinding, mixing, melting, and emulsifying one or more types of cheese, with or without the addition of milk ingredients, sometimes with the addition of other food components, contain roughly the same proportions of nutrients as the cheeses from which they were made [1].

Cheese is a complex matrix and different factors, e.g., pH, temperature, water activity, and microflora throughout ripening influence its chemical features [4]. It contains a substantial amount of important nutrients, such as: Proteins, bioactive peptides, amino acids, fat, fatty acids, vitamins, and minerals, which makes it an energy-rich and nutritious food [2]. Nowadays the main reasons for the consumption of cheese are the supply of important and essential nutrients, its manifold uses in cooking and meal preparation, as well as enjoyment [2]. The consumer’s acceptance of cheese mainly depends on its sensory attributes and flavor is considered as the most important quality criteria of fresh and aged cheese [5]. The flavor of mature cheese is the result of a series of biochemical changes that occur in the curd during ripening, caused by the interaction of starter bacteria, enzymes from the milk, enzymes from the rennet, accompanying lipases, and secondary flora. It is established that flavor of cheeses manufactured from raw milk is more intense than the flavor of cheeses produced from pasteurized or heat-treated milks, which results for high levels of native lactic acid bacteria present in raw milk [4]. During ripening period of cheese complex biochemical and chemical processes, lipolysis, proteolysis and fermentation occur and cheese undergoes significant changes which give rise to the typical flavor, texture, and appearance associated with particular type of cheese [6]. Biochemical changes in cheese during ripening may be grouped into primary (lipolysis, proteolysis and metabolism of residual lactose and of lactate and citrate) or secondary (metabolism of fatty acids and of amino acids) events [7]. During processing and ripening, a wide range of volatile compounds, of different polarity and reactivity arises. They are involved in cheese aroma and flavor [5]. These compounds derived from three major metabolic pathways: catabolism of lactate, protein and lipid. Lipolysis is one of important biochemical event occurring during cheese ripening which results directly in the formation of flavor compounds by liberating free fatty acids (FFA), which may directly contribute to cheese flavor and also serve as substrates for further reactions producing highly flavored catabolic end products, such as alkan-2-ones and fatty acid lactones [6,8]. In cheeses where lipolysis reaches high levels (e.g., blue and hard Italian cheese (ITA)) it is a major pathway for flavor generation. However, if levels of lipolysis are moderate during ripening, the contribution of its final products to cheese quality and flavor has received relatively little attention (e.g., cheddar and gouda) [8]. Microbiological changes during ripening of cheese include the death and lysis of starter cells, the growth of an adventitious flora (‘nonstarter lactic acid bacteria’, principally facultatively heterofermentative Lactobacilli) and, in many varieties, the development of a secondary microflora, e.g., *Propionibacterium freudenreichii* in Swiss cheeses (SUI), molds in mold-ripened varieties and a complex Gram-positive bacterial flora in smear cheeses, which is often of great importance to the flavor and, in some instances, the texture of these varieties [7]. Ripening cheese can be a good source of probiotic lactic acid bacteria, as the survival of probiotic cultures in ripening cheeses is greater comparing to other, more acidified dairy products [9].

Cheese is easily digestible and rich in nutritional compounds, especially proteins, fatty acids (FA), vitamins and minerals, especially calcium. It is also an important source of a wide variety of biologically active substances among which specific FA are of utmost importance. Milk and dairy fat is the most complex fat in the human diet and consists of more than 400 distinct FA. FA composition of cheese varies according to milk origin (e.g., species and breed), rearing conditions (e.g., feeding and management), and cheese-making technology (e.g., coagulation process, addition of salt, ripening period) [10]. Conjugated fatty acids (CFA) are bioactive isomers present in rumen animal-derived milk and meat, which are widely studied because of numerous health-beneficial properties and interesting biological functions [11]. Among them conjugated linoleic acids (CLA) and especially c9t11C18:2 (rumenic acid; RA) are important because of their wide health-promoting biological activities, which were studied in vitro, and in vivo in animal models and human studies [12]. Also other CFA can be present in cheese fat because of the activity of ruminal microbiota [11] as well as the activity of bacterial flora present in cheese, as different strains of lactic bacteria are able to increase CFA content in food products [13,14]. Odd- and branched-chain fatty (OCFA and BCFA) are another important group of specific FA. They are mainly saturated fatty acids (SFA) with one or more methyl branches in the *iso*- or *anteiso*-position, which are largely derived from ruminal bacteria [15]. They are transferred to ruminant tissue and became an integral constituents of milk and meat. The results of research show that OCFA and BCFA have specific health-promoting properties, e.g., anti-cancer activity [15,16]. Microorganisms inhabiting rumen also are able to release phytol from chlorophyll, after which phytol is converted into phytanic acid (3,7,11,15-methylC16:0), which is subsequently metabolized into pristanic acid (2,6,10,14-methylC15:0) [17,18]. Diet is a main source of phytanic acid, in particular ruminant fats, fish and dairy products are rich sources of phytanic acid [19]. Due to the ruminal microorganisms activity dairy fat is also the richest dietary source of natural *trans* FA isomers, mainly vaccenic acid (trans11C18:1, VA) indicating quite opposite, beneficial properties, in comparison to artificial *trans* FA of partially hydrogenated oils [20]. 

Because of such a potential great variety of bioactive FA in cheese, the aim of present study was to determine the FA profile of commercially available ripening cheeses, with the special emphasis on CFA profile. Different type of ripening cheese meant to be characterized in terms of FA content in comparison with curd cheeses (CC), as the most similar to milk fat, and with processed cheeses (PC) of diversified and unsettled/unspecified origin. Chemometric is a science of multidisciplinary nature which involves multivariate statistics, mathematical modelling and information technology, specifically applied to chemical data [21]. Chemometric methods have been previously used in evaluating the quality and identity control of processing parameters for dairy products [22,23,24,25,26,27] but there is still lack of data of FA and CFA profiles investigation. Multivariate analyses of chromatographic data have been chosen to represent a powerful method for discrimination between types of cheeses. Chemometric approach has been proposed in this study as an objective approach for the evaluation and data interpretation of FA profiles and CFA profile in different types of ripening cheeses in comparison to CC and PC. 

## 2. Results

### 2.1. Fat Content

**Figure 1:**
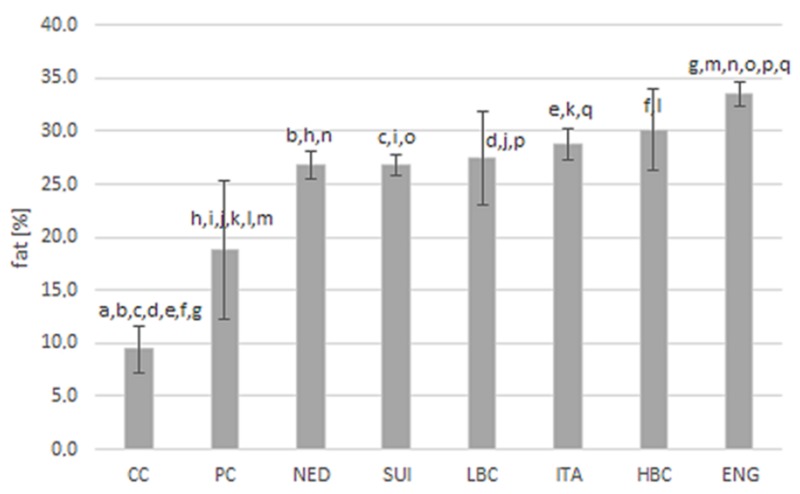
Fat content (%) in examined types of cheese. CC—Curd cheese, PC—Processed cheese, NED—Dutch type cheese, SUI—Swiss type cheese, LBC—Lichen blue cheese, ITA—Italian type cheese, HBC—Hypertrophied blue cheese, ENG—English type cheese; Values sharing a letter (a–q) are statistically different; *p*-value ≤ 0.05 was considered significant.

Fat content in examined types of cheese is presented in Figure 1. The highest amount of fat (33.5 ± 1.2%) was quantified in ENG cheeses, which significantly exceeded the content in CC and PC and in most of ripening cheeses (except HBC). Level of fat in CC (9.4 ± 2.2%) and in PC (18.8 ± 6.5%) was significantly lower than in all ripening cheeses.

### 2.2. Saturated Fatty Acids Content 

**Figure 2:**
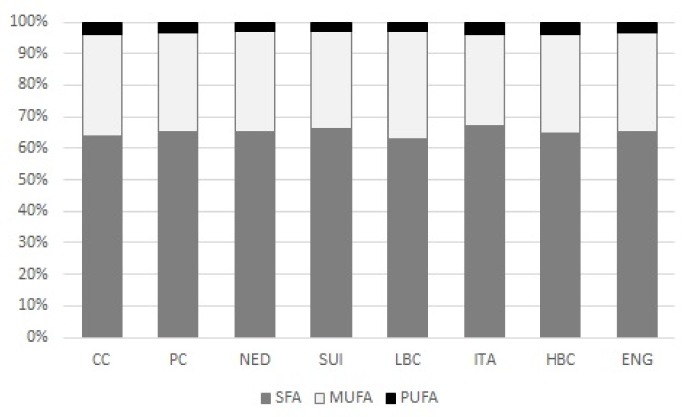
Mean percentage share of saturated (SFA), monounsaturated (MUFA) and polyunsaturated (PUFA) fatty acids in total FA pool in examined types of cheese. CC—Curd cheese, PC—Processed cheese, NED—Dutch type cheese, SUI—Swiss type cheese, LBC—Lichen blue cheese, ITA—Italian type cheese, HBC—Hypertrophied blue cheese, ENG—English type cheese, SFA—Saturated fatty acids, MUFA—Monounsaturated fatty acids, PUFA—Polyunsaturated fatty acids; mean percentage share was calculated as the ratio of the fatty acid content of a given group (SFA, MUFA, or PUFA, respectively) to their total combined content (SFA + MUFA + PUFA) considered as 100%.

SFA predominated in all examined types of cheese constituting >60% of all FA (Figure 2). Their significantly lower amounts (expressed in µg/g of cheese) than in ripening cheeses were detected in CC and PC (Table 1). Applied GC-MS method enabled the measurement of the content of 25 SFA (Table 1) with 10 FA of branched carbon chain (BCFA). Only contents of C11:0 and i-C14:0 did not differ among investigated types of cheese whereas contents of remaining 23 SFA significantly differ. 

Palmitic acid (C16:0) predominated in SFA and its highest amount was detected in ITA. Content of C16:0 in all types of ripening cheeses exceeded its content in CC and PC. Among ripening cheeses its significantly lower level than in ITA was detected in LBC and ENG. Amount of miristic acid (C14:0) also was the highest in ITA, whereas its lowest content was detected in CC and PC and in case of ripening cheeses, in LBC and ENG. CC differed from all ripening cheeses in terms of C14:0 amount and PC also differed from ripening cheeses (except LBC). Content of C14:0 in ITA, SUI and HBC significantly exceeded its content in CC, PC, and LBC and C14:0 content in ITA also predominated its content in ENG. Stearic acid (C18:0), the third most abundant SFA, was detected in highest content in HBC. However, in all ripening cheeses its levels exceeded those in CC and PC. In case of medium chain FA of even numbers of carbon, amounts of C6:0 in ripening cheeses were significantly higher than in CC and PC, with the highest content in ITA, which also exceeded C6:0 content in LBC and ENG. Similar tendency was observed for C8:0, and its high content was also detected in SUI, which predominated content in LBC and ENG. Among ripening cheeses, the highest levels of C10:0 were observed in ITA, SUI and HBC. They significantly exceeded not only those in CC and PC, but also in LBC and ENG (except of HBC). Lauric acid (C12:0) was detected in the highest amounts in ITA and SUI which exceeded its content in LBC, CC, and PC. Long chain saturated fatty acids were present only in trace amounts. Their content seemed to decrease with the increase on carbon chain length. C24:0 only in ENG was present in higher amount than in other examined types of cheese. Higher levels of C22:0 were detected only in LBC and ENG. In case of C20:0 its lower content than in ripening cheeses was quantified in CC. Its highest amount was determined in NED and it exceeded not only content in CC but also in PC.

Among SFA five odd-chain FA were also quantified. Content of C11:0 did not differ among examined types of cheese. In case of C9:0 its content in SUI was residual and the highest content was detected in ITA. CC contained significantly less of C13:0 than ripening cheeses. Its level in PC was twice as high as in CC but still it was significantly less that in ITA, SUI, and HBC, where its content was the highest. Among ripening cheeses amounts of C13:0 were diversified. Significantly higher content was detected also in ITA, SUI and HBC in comparison to LBC, ENG, and NED (except HBC). C15:0 in all ripening cheeses exceeded its content in CC and PC. Its highest level was detected in ITA, which was significantly higher than in LBC and ENG. In case of C17:0 significant differences were observed only among CC and PC and all types of ripening cheeses.

Branch-chain FA included both *iso*- (i-) and *anteiso*- (a-) FA isomers. All ripening cheese were more abundant in a-C16:0, which predominated in BCFA, than CC and PC. Among ripening cheeses significant differences in levels of this FA were observed between ITA, with its highest content, and LBC. a-C15:0 was present in high amounts. Similarly, as in the case of other FA, CC and PC were less abundant in this isomer than ripening cheeses. However, among ripening cheeses also some differences were observed, e.g., a-C15:0 in ITA and SUI significantly exceeded its content in LBC. Iso-FA were present in lower amounts than *anteiso*-FA, and in this group of FA i-C16:0 predominated. Its lowest level was detected in CC and the highest—in SUI. Content of i-C16:0 in SUI significantly exceeded its amounts not only in CC and PC, but also in ENG and LBC. ENG and LBC was the less abundant in this isomer of all ripening cheeses. In case of i-C17:0 its level in CC was significantly lower than in ripening cheeses. Its highest content was detected in HBC. Isomer i-C15:0 was present in significantly lower amounts in CC and PC than in ripening cheeses. Among ripening cheeses content of i-C15:0 in SUI and ITA significantly exceeded its content in LBC. No significant differences were observed only for i-C14:0 among examined types of cheese, however similar tendency for its lower content were found for CC and PC. Similar levels were quantified for i-C13:0 and i-C18:0. Significant differences among examined types of cheese in case of i-C13:0 were observed between SUI of the highest content and CC, PC, LBC, HBC, and ENG. Also NED was more abundant in this FA than CC, PC and LBC. In case of i-C18:0 differences were observed between CC and PC and LBC and HBC. Interesting observations considered 2,6,10,14-methylC15:0 and 3,7,11,15-methylC16:0, which content in ENG significantly exceeded their content in almost all of other examined types of cheese.

### 2.3. Monounsaturated Fatty Acids Content 

Number of quantified MUFA equaled the number of SFA, as 25 different MUFA were detected in examined cheeses (Table 2). MUFA constituted about 30% of total FA content (Figure 2). CC and PC were the less abundant in MUFA and their content was significantly lower than in ripening cheeses. Although the highest level of MUFA was quantified in ITA, no differences were observed among ripening cheeses. In total MUFA predominated *cis* isomers, but also 7 different *trans* isomers were detected: one isomer of C16:1, two isomers of C17:1 and four isomers of C18:1.

In total MUFA pool C18:1 *cis* isomers predominated, especially c9C18:1, whose content highly exceeded the content of other MUFA. As c9C18:1 mainly determines total MUFA content, its significantly higher amounts were detected in ripening cheeses, especially in ITA and SUI, in comparison to CC and PC. The second most abundant MUFA was c9C16:1 and its significantly higher content was detected in ripening cheeses. Within ripening cheeses, the highest content of c9C16:1 was determined in ITA, which significantly exceeded its content in LBC. In case of c7C16:1 its content in ripening cheeses also significantly exceeded its levels in CC and PC and within ripening cheeses significant differences were observed only between the types of the highest (SUI) and the lowest (LBC) content of c7C16:1. Content od c9C14:1 was almost as high as c9C16:1, and it was significantly highest in ripening cheeses in comparison to CC and PC. Among ripening cheeses significant differences were observed among types of the highest (ITA and SUI) and the lowest (LBC) content. Two C12:1 isomers of comparable content were also quantified. CC and PC contained their less amounts than most of ripening cheeses. Within ripening cheeses similarly low content of c9C12:1 and c11C12:1 as in CC and PC, was detected in LBC. The highest amounts of these C12:1 isomers were detected in ITA and they were significantly highest than in CC, PC and LBC (in case of c9C12:1) and in CC and PC (in case of c11C12:1). For C10:1, which was shortest of all MUFA, position of double bond was not established. Its significant content in ITA and SUI exceeded its content in CC, PC and LBC. c9C17:1 predominated in SUI. Similarly, as in other cases, its amount in ripening cheeses exceeded its level in CC and PC. For LBC significant difference in c9C17:1 content was observed for CC. Nine different positional isomers of C18:1 were detected. Beside c9C18:1, quit high amount was also quantified for c6C18:1. Its content in ripening cheeses significantly exceeded its content in CC and PC. Within ripening cheeses its level in ENG was significantly higher than in ITA. In case of c7C18:1 some differentiation in its levels was observed. The lowest content, significantly different than in other types of examined cheeses, was detected in CC. The highest amount was quantified in ITA, which exceeded its content in CC, PC, NED, LBC, and ENG, and in HBC, which exceeded its content in CC, PC, NED, and ENG. Isomers c10C18:1 and c13C18:1 in ripening cheeses were present in comparable amounts, only in CC and PC their contents were significantly lower. In case of c11C18:1, except significantly lower content in CC and PC, some differences in its amounts were also observed among ripening cheeses. Its highest content in HBC exceeded its content in NED, LBC and ENG. Moreover, its content in ITA and SUI was significantly higher than in ENG. CC contained significantly less amount of c12C18:1 than ripening cheeses, except ENG. In case of PC differences in c12C18:1 content were observed only with ITA and HBC of the highest content of this isomer. c12C18:1 content in ITA differed also from its content in some ripening cheeses: NED, LBC, and ENG. Similarly, in case of HBC its content of c12C18:1 was higher than in LBC and ENG. Levels of c14C18:1 in CC and PC differed significantly from all (CC) or most of (PC) ripening cheeses. Within ripening cheeses predominating content of c14C18:1 in ITA and HBC exceeded its levels in ENG. Content of c15C18:1 was only vestigial in selected types of cheese (PC, LBC, and ITA) and in other examined cheeses its amounts were diversified with the highest detected in HBC. Content of c9C20:1 in selected ripening cheeses (NED, SUI, LBC, ITA, and HBC) significantly exceeded its level in CC. In case of c11C20:1 significant differences were observed among CC and NED and CC and ITA, the last of the highest content. 

In case of *trans* isomers of MUFA t7C17:1 was present in trace amounts in almost all examined cheeses, except of ENG. The highest levels of t9C17:1 were determined in HBC, NED, and ENG, whereas in other types of cheese its amounts were vestigial. Significantly higher amounts were quantified for t9C16:1, whose highest levels were determined in NED and the lowest—in CC. Its content in NED and ENG exceeded its levels in CC, PC and ITA. Significant differences were observed also for HBC and CC. Among four *trans* isomers of C18:1, the highest content was observed for VA (t11C18:1). Its amounts in CC and PC were lower than in ripening cheeses whereas the highest level was quantified in HBC. Content of t11C18:1 in HBC exceeded its contents not only in CC and PC, but also in NED, LBC and ENG. Amounts of t9C18:1 in CC and PC differed significantly from its levels in ripening cheeses. Similar tendency was detected for t8C18:1 but in case of this isomer greater diversity of its levels in ripening cheeses was observed. Its content in SUI and HBC significantly exceeded its content in ENG. Isomer t6C18:1 was present in CC and SUI in trace amounts and in other examined types of cheese its content did not differ. 

### 2.4. Polyunsaturated Fatty Acids Content 

Percentage share of PUFA in total FA pool was the lowest (Figure 2). Content of 14 PUFA was determined whereas conjugated C18:3 were detected only in ENG and HBC (Table 3). The highest content of PUFA was detected in ITA and it exceeded the content CC, PC, NED, LBC and ENG. HBC were abundant in PUFA where their level was significantly higher than in CC, PC and LBC. In ITA predominated n3PUFA and n6PUFA, but the highest n6PUFA content detected in ITA, significantly larger than in CC, PC, NED, LBC, and ENG resulted in the lowest n3/n6 ratio in ITA. Similar dependence was observed for HBC. The highest values of n3/n6 ratio were established for NED and ENG, which significantly differed from its values for ITA and HBC. CC and PC were the poorest sources of n3PUFA and all ripening cheeses significantly exceeded n3PUFA content in CC and PC. Six different C18:2 isomers were detected in examined samples of cheese: Four unconjugated and two conjugated. Linoleic acid (c9c12C18:2; LA) predominated in all types of cheese with ITA and HBC being its richest sources. Its content in ITA and HBC significantly exceeded its amounts in most of other types of cheese, except SUI. Two geometric isomers of c9c12C18:2 were detected: t9t12C18:2 and t9c12C18:2. The lowest contents of both of them were quantified in CC and t9t12C18:2 content differed significantly from its amounts in most of ripening cheeses (except LBC) whereas t9c12C18:2 levels in ripening cheeses significantly exceeded its contents in CC and PC. Similar tendency was observed for t11c15C18:2 (vaccelenic acid), which is positional isomer of C18:2 detected in cheeses. Its small amount in CC differed significantly form all of ripening cheeses. Its content in PC, was significantly lower than in NED, ITA and HBC. However, in both CC and PC big differences in t11c15C18:2 content were observed. Two conjugated isomers of C18:2 were detected: c9t11C18:2, and CLA isomer of two *trans* bonds. The amounts of the second one were diversified within examined types of cheese. The highest content of ttCLA was detected in ITA and it exceeded significantly the amounts of this isomer in CC, PC, SUI, LBC and ENG. RA content was the lowest in CC and it differed significantly from all ripening cheeses. In PC amount of c9t11C18:2 was significantly lower than in NED, SUI, HBC, and ENG. The most abundant in RA were ENG, where its content significantly exceeded the amounts of this isomer in CC, PC, LBC, and ITA. An α-linolenic acid (c9c12c15C18:3; ALA) predominated in ITA where its content significantly exceeded the amounts quantified in CC, PC, and LBC. The poorest sources of this n3 FA were CC and PC, which differed significantly form all ripening cheeses. The most abundant in c4c7c10c13c16c19C22:6 were NED where its content significantly exceeded small levels detected in CC, PC, SUI, LBC, and ITA. In case of c6c9c12C18:3 the highest content, significantly greater than in other examined types of cheese, was detected in HBC whereas in PC, SUI, and ITA only trace amounts of this n6 FA was determined. Level of another FA of n6 family—c8c11c14C20:3 was the highest in ITA and in CC, PC, and SUI its content was vestigial. As far as c5c8c11c14C20:4 is concerned, its content in NED, ITA, and SUI was significantly higher than its levels detected in CC, PC, and LBC.

### 2.5. The Content of Conjugated Fatty Acids (CFA)

**Figure 3:**
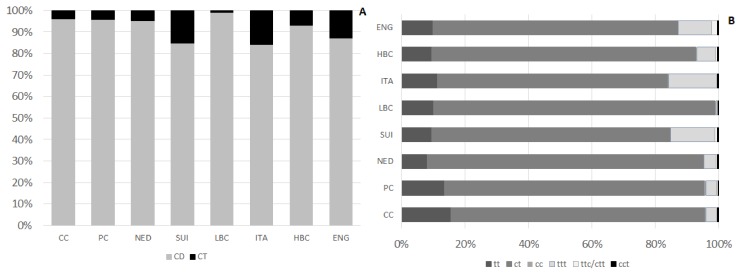
Percentage share of CFA in examined types of cheese: CD and CT share (**A**) and share of different types of isomers (**B**). CC—Curd cheese, PC—Processed cheese, NED—Dutch type cheese, SUI—Swiss type cheese, LBC—Lichen blue cheese, ITA—Italian type cheese, HBC—Hypertrophied blue cheese, ENG—English type cheese, CFA—Conjugated fatty acids, CD—Conjugated dienes, CT—Conjugated trienes, c—*cis*, t—*trans*.

HPLC analysis carried out on four ion-exchange columns loaded with silver ions allowed to quantified CFA differing with number of double bonds (two or three) and conformation (*cis* and *trans*) isomers. CFA content is presented in Table 4. Their content differed significantly among examined types of cheese. The lowest amount of CFA, significantly different than in ripening cheeses, was detected in CC and PC. The highest CFA level, significantly exceeding their content in cheeses of other types, was observed in ENG. Beyond ENG, no other types of ripening cheeses varied in terms of CFA content. CD, which predominated in CFA, constituted from 84.4 ± 5.5% to 99.1 ± 1.4% in LBC of CFA (Figure 3A). They amounted from 383 ± 133 µg/g of cheese in CC to 1513 ± 342 µg/g of cheese in ENG. CD level in CC was significantly lower than in ripening cheeses. Similarly, CD level in PC was lower than in most of ripening cheeses, except NED and ITA. Mean CD content in ITA and NED, although exceeded their content in CC, was significantly lower than in ENG. Among CD three groups of isomers differing with bonds geometry were distinguished: tt (*trans*, *trans*), ct (*cis*, *trans*), and cc (*cis*, *cis*) isomers (Figure 3B). Amount of ct isomers ranged from 320 ± 115 µg/g of cheese in CC to 1342 ± 310 µg/g of cheese in ENG. Differences in ct content were observed among CC and PC and ripening cheeses. Within ripening cheeses substantial differentiation in ct content was detected, as their amount in ENG exceeded significantly ct content in other types of ripening cheeses. Among ct isomers of CD c9t11CLA (RA) was the main quantified isomer, constituting from 73.1 ± 3.8% in CC to 80.5 ± 2.4% of all quantified CD in NED. Concomitantly c9t11CLA constituted from 63.7 ± 19.9% in ENG to 76.8 ± 5.0% of CFA in LBC. RA content in CC was significantly lower than in ripening cheese. In PC its amount was almost twice as much as in CC and in ripening cheeses RA content >700 µg/g of cheese. The richest source of c9t11CLA were ENG and its content in ENG exceeded significantly RA levels in CC, PC, NED, and ITA.

Isomers with two conjugated double bonds of tt conformation were detected in the lowest amounts in CC and then successively in NED and PC. Ripening cheeses (except NED) were significantly richer in tt than CC whereas NED were significantly less abundant in tt than SUI, ITA, HBC, and ENG.

Among CD, the lowest content was found for cc isomers. Their content in PC, NED, and ENG was residual, <0.01. The highest amounts of these CD isomers were detected in ITA and HBC, however in ITA their content was diversified.

CT content in examined cheeses was far less than CD. Their lowest amount was found in LBC, which was significantly lower than in HBC, ITA, SUI, and ENG. Among CT group of isomers with three double bonds of *trans* configuration predominated and in ENG, ITA, and SUI their content significantly exceeded ttt content in other types of examined cheeses. ENG were significantly more abundant in ttc/ctt isomers than cheeses of other types. Quite high level of ttc/ctt was also quantified in SUI, whereas in CC their amount was the lowest. Isomers of cct group were the less abundant than other CT and their content was diversified among different brands of cheese within one type, which resulted in lack of significant differences in cct content among examined types of cheese.

### 2.6. Cluster Analysis (CA)

**Figure 4:**
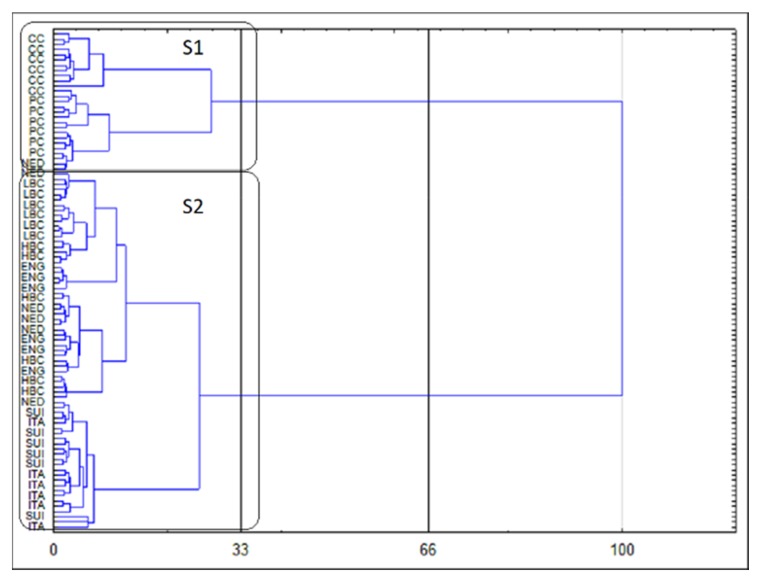
Dendrogram of similarities in fatty acids (FA) and conjugated FA (CFA) profile of investigated samples of cheese.

The results of CA are presented as a dendrogram in Figure 4. The application of the less restrictive Sneath’s criterion (66%) as well as the rigorous Sneath’s criterion (33%) to the dendrogram analysis allowed to distinguish two clusters (S1 and S2), that differentiate the examined samples. The first cluster (S1) included samples of CC and PC, whereas samples derived from ripening cheeses were incorporated into second cluster (S2). Further analysis of the dendrogram indicated a clear separation of CC samples and PC samples in S1 cluster. Similarly, within S2 cluster, ITA and SUI samples were separated from remaining matured cheeses samples, but these observations are below the Sneath’s criterion enabling the assessment of the number of significant clusters.

### 2.7. Principal Component Analysis (PCA)

PCA analysis identified 66 factors, of which the first four were selected for further analysis, carrying a total of 72.5% of the total variability (Figure S1). 

**Figure 5:**
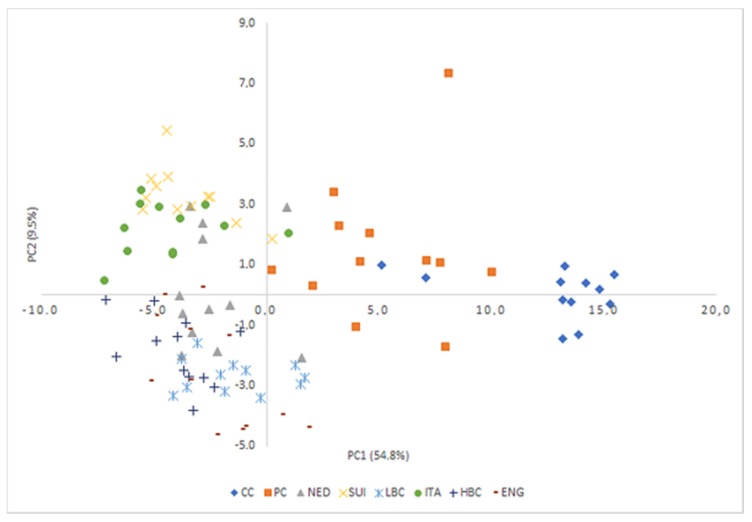
Projection of cheese samples on the plane of the principal components. CC—Curd cheese, PC—Processed cheese, NED—Dutch type cheese, SUI—Swiss type cheese, LBC—Lichen blue cheese, ITA—Italian type cheese, HBC—Hypertrophied blue cheese, ENG—English type cheese.

The detailed matrix of factor analysis structure with the factor load values is presented in Table S1. The two first Principal Components explained 64.3% of the total variance (PC1: 54.8% and PC2: 9.5%, respectively). Statistical tests revealed the existence of significant differences between the values of all four principal components among the experimental groups (Table 5), which is also observable in the projection of objects on the plane of the principal components (Figure 5). There was a fair separation of samples depending on their variety in the projection of PC1. PC1 is the main responsible for the discrimination between CC and PC from ripening cheeses, as CC and PC samples were located at positive values of PC1, while ripening cheese samples were located in negative values of this principal component. In turn, the separation of individual ripening cheeses was noticeable on the PC2 plane, with ITA and SUI clearly separating from other ripening cheeses, especially ENG HBC and LBC. PC2 allows the discrimination between CC and PC and most of the ripening cheeses (LBC, ENG and HBC with negative scores, SUI and ITA with high positive scores and NED with low positive scores). 

### 2.8. Linear Discriminant Analysis (LDA)

LDA was used to obtain appropriate classification rules for examined cheese samples. Relevant discriminant functions were calculated in a stepwise progressive method as linear combinations of selected 39 variables with the highest factor load in individual factors (over 0.8) recognized in PCA. In selected method, initially all variables were outside the model. In the next steps the variables with the highest discriminant value, according to the Wilks’ λ statistic test, were included in the model. In performed analysis, 22 variables have been included in the final model. Applied canonical analysis allowed to distinguish 3 statistically significant discriminant functions. DF1 is the most significant function, as it explains over 62% of discriminatory power. The DF2 and DF3 explain only 15% and 10% of discriminatory power respectively (Table 6).

**Figure 6:**
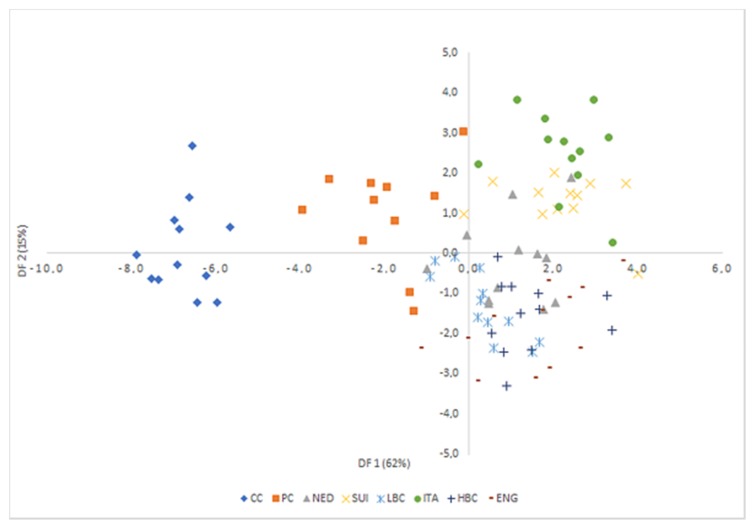
Scatterplot of canonical values for functions DF1 and DF2. CC—Curd cheese, PC—Processed cheese, NED—Dutch type cheese, SUI—Swiss type cheese, LBC—Lichen blue cheese, ITA—Italian type cheese, HBC—Hypertrophied blue cheese, ENG—English type cheese.

**Figure 7:**
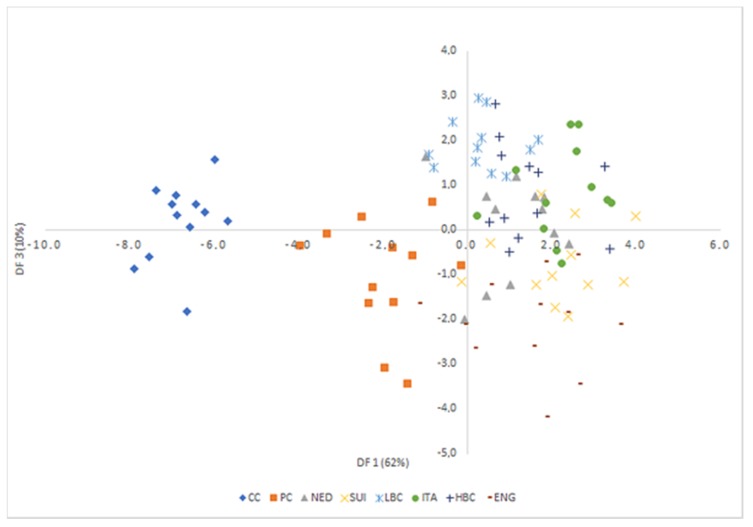
Scatterplot of canonical values for functions DF1 and DF3. CC—Curd cheese, PC—Processed cheese, NED—Dutch type cheese, SUI—Swiss type cheese, LBC—Lichen blue cheese, ITA—Italian type cheese, HBC—Hypertrophied blue cheese, ENG—English type cheese.

**Figure 8:**
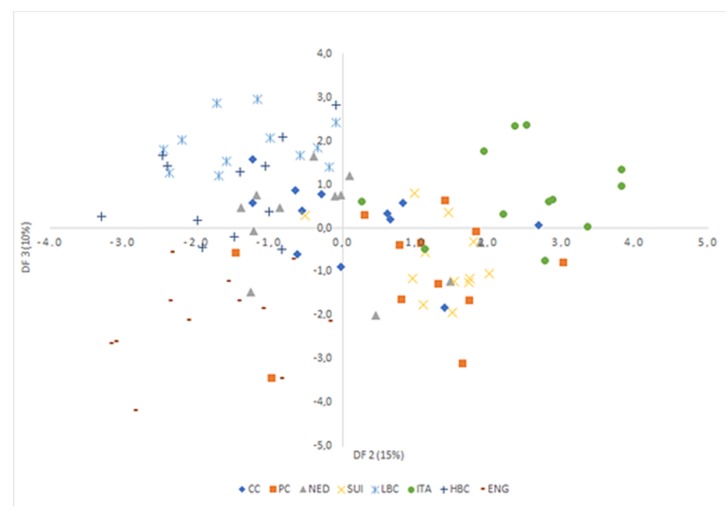
Scatterplot of canonical values for functions DF2 and DF3. CC—Curd cheese, PC—Processed cheese, NED—Dutch type cheese, SUI—Swiss type cheese, LBC—Lichen blue cheese, ITA—Italian type cheese, HBC—Hypertrophied blue cheese, ENG—English type cheese.

Analysis of canonical mean variables indicated that DF1 had the greatest impact on the distinction of all samples, with particular emphasis on those of CC and PC cheeses. DF2 seemed to distinguish mostly matured cheeses: ITA from LBC, HBC, and ENG, while DF3 differentiated mostly samples from LBC and ENG. Graph analysis confirms the suggestions provided by the values of average canonical variables (Figure 6, Figure 7 and Figure 8).

The calculated classification matrix indicated that the average classification efficiency based on the calculated functions was 86.5% (Table 7). For individual groups these coefficients were as follows: 100% for CC, LBC and ITA samples, 83% for PC, SUI, and ENG, 75% for NED and 66% for HBC.

## 3. Discussion

Milk and dairy products are not only a rich sources of essential nutrients (protein, calcium and vitamins) but also contain numerous bioactive molecules, among which FA are of utmost importance. Fat of dairy products consists of more than 400 distinct FA, which makes it the most complex fat of all present in human diet. Moreover, many of these FA are derived in our diets in significant amounts almost exclusively from dairy products [20]. Both milk and dairy products are important sources of C12:0, C14:0, C16:0, and total SFA in Western diet, but also contain other FA with beneficial influence on onset and development of chronic disease, such as: 4:0, several OCFA and BCFA, c9C18:1, and RA [28]. There are several factors, such as the diet, stage of lactation, health, and breed of lactating females which may influence FA composition of milk and subsequently—FA profile of dairy products. It was established, that FA profile of milk and dairy products can affect human health and feeding systems based on herbage, especially highland pasture, have been shown to yield milk and dairy products with FA profiles more beneficial to human health [29]. Cheese composition depends on the milk’s microbiological and chemical composition, the cheese-making technology, ripening time, and cheese factory conditions [10]. Milk fat and especially FA profile are also essential for sensory properties and the development of correct flavor in cheese during ripening. Fat is not only a source of flavor compounds but also a solvent for fat-soluble flavor compounds and provider of fat–water–protein interface for flavor forming reactions [8]. The principal lipids of milk are triacylglycerides, which may represent up to 98% of the total lipids. Positioning of FA in triacylglycerides is non-random. Short-chained FA (e.g., C4:0 and C6:0 are predominately located at the sn-1 and sn-3 positions, and with the increase of chain length up to C16:0 an increasing proportion is esterified at the sn-2 position. C18:0 is generally located at the sn-1 position, while unsaturated fatty acids are esterified mainly at the sn-1 and sn-3 positions [8].

Milk fat, which is characterized by a high level of SFA and low level of unsaturated FA, especially n3PUFA, reflects ruminal biohydrogenation of PUFA and *de novo* mammary synthesis of SFA [30]. Bergamaschi et al. found the FA profile was affected by cheese ripening, especially during the first phase from 0 to 6 months, as they observed an increase in medium chain SFA and a decrease in many PUFA [29]. In our study percentage share of SFA, MUFA, and PUFA did not differ among ripening cheeses but there were significant differences in their total content as well as in the content of most of examined individual FA. ITA and HBC were the most abundant in PUFA, both in n6PUFA and in n3PUFA, which resulted in the lowest n3/n6 ratio in these types of ripening cheese. However, in all examined types of cheese this ratio was relatively small (<0.3), which confirms that dairy fat cannot be considered as important dietary source of favorable PUFA. RA and other CLA isomers of substantial content in cheese are accounted as the most valuable health-promoting PUFA.

It has been established, that increased consumption of dairy products had an association with an increase in blood plasma OCFA, especially C15:0 and C17:0 which can be utilized as rough markers for dairy fat intake. It was demonstrated, that higher plasma concentrations of OCFA are associated with lower risk of cardiovascular diseases (CVD), although the mechanism responsible for this is still debated [31]. Both C15:0 and C17:0 were present in higher amounts in matured cheeses, especially in ITA, which makes their FA profile more favorable. OCFA and BCFA were also studied in terms of obesity and decreased serum levels of *iso*-BCFA, which correlated with increased levels of triglycerides [32]. In examined types of ripening cheeses *anteiso*-FA exceeded *iso*-FA however in ITA and SUI BCFA were present in high amounts. ENG were the richest sources of phytanic acid and pristanic acids, which are considered as pasturing indicators but also as health-promoting FA [17,18,19,33]. In this type of ripening cheeses also CD and CT of CFA predominated, with the highest content of RA of highly recognized advantageous biological activity, which makes ENG valuable dietary sources of active FA [12,34].

Although several earlier surveys was focused on FA composition of cheeses grouped according to the ruminant species of the milk used in manufacturing: sheep, goat, and cow [35,36,37,38]; as well as of traditional cheeses registered as PDO (protected designation of origin) or PGI (protected geographical indication) [10,21,39,40], studies concerning the evaluation of FA profile of commercially available chesses of different types and groups, are still scarce [41,42,43]. That is why in the present study highly sophisticated analytical procedures (both instrumental and statistical) was combined for the examination of commercially available ripening cheeses. Taking into account the wide variety of this dairy products on the market, we have decided not to limit our choice to the selected types of cheese, but to reflect as big part of assortment of these foodstuffs as possible. Although there are different types of cheese classification [44], we have decided to classify ripening cheeses in the simplest way, according to their place of origin (ITA, SUI, NED, ENG) or according to the type of mold (LBC and HBC). This type of classification in accessible but also the most convenient for regular consumers.

To achieve complete characterization of FA profiles in milk fat, with a special emphasis on CFA, suitable analytical techniques need to be applied. Various chromatographic methods coupled with mass spectrometry detection have been reported for lipid analysis in milk samples, including mainly GC–MS and LC-MS methods, which are suitable for FA profiling [33,34,35]. However, they cannot provide comprehensive CFA profile information with sufficient sensitivity to quantify these components in dairy fat. In present study we used Ag^+^-HPLC-DAD method for CFA profile analysis and their quantification in different types of ripening cheese. This attempt not only gave more comprehensive information about the profile of these bioactive FA in different types of cheese, but also contributed to their precise identification with chemometric tools. As CFA are unique group of FA their detailed profile may serve as indicator of originality, traceability and other quality characteristics of ruminant’ derived foods. That is why in present study not only general FA profile was evaluated but also special emphasis was put into the CFA profile of dairy products. In our opinion such comprehensive overview of FA composition will provide a solid basis for application of various chemometric tools to achieve reliable results. This also may be considered as significant aspect of novelty in our research. To our best knowledge, the current study is the first attempt of application of Ag+-HPLC-DAD analysis carried out on four ion-exchange columns loaded with silver ions used in conjunction and photodiode array detection to evaluation of free (underivatized) FA content in dairy products. The applied HPLC method for identification and quantification of free CFA may be considered as a complementary method to the most commonly used GC-FID and GC-MS ones. It can be successfully utilized for analysis of quality, authenticity and traceability of milk and dairy foodstuffs, especially those endangered of adulteration with other fats of a lower nutritional value (mainly butter and cheeses).

Wide spectrum of multivariate methods is available in order to extract information from the data sets obtained from cheese analyses. Many authors used them to evaluate data of volatile compounds profile, content of casein, peptides, amino acids, sensory determinants, etc. and they obtained successful classification or differentiation of examined samples [21,23,24,25,26,45]. In the present study, chemometric analyses were successfully applied to large data sets of FA and CFA content in ripening cheeses of different types, fresh cheeses (crud cheeses) and processed cheeses. In CA analysis of total FA and CFA profile caused clear separation of CC and PC from ripening cheese, which indicates great similarity in FA and CFA profiles of these two types of cheeses. As FA and CFA content was expressed in relation to sample, not in relation to fat, observed differences partly resulted from the significantly lower fat content in CC and PC.

Principal components analysis can be used to identify patterns and to explain similarities and differences in data, as well as to show relationships that exist between objects and arbitrary principal components [42]. The two methods CA and PCA showed similar grouping of cheese samples according to FA and CFA content. However, PCA was successfully used not only for the distinguishing of CC and PC from ripening cheese but also enabled excretion of ITA and SUI from other matured cheeses. PC1 contributed 54.8% and was composed primarily of 44 of analyzed variables (Table S1). PC2 accounted for only for 9.5% and was composed of C20:0, sum of FA, C11:0, phytanic acid and pristanic acid. Classification of individual ripening cheeses was noticeable on the PC2 plane, with ITA and SUI clearly separated from other ripening cheeses, especially ENG, HBC and LBC, which was easily interpreted using appropriate plots. In the PCA plot with PC1 and PC2 (Figure 5) these three distinct groups were identifiable.

In LDA only 22 variables have been included in the final model, which allowed to distinguish 3 statistically significant discriminant functions. Average classification efficiency based on the calculated functions was quite high and equaled 86.5%. In summary, the applied LDA allowed to observe significant differences among all groups of samples which indicated that the analysis of FA profile allowed for the distinction of the origin of the cheese sample with fair probability. The FA composition of cheese elaborated with various chemometric methods can be used to some extent to differentiate the milk of animal species from which the cheese is made, the geographical origin of product and thus, provide information about the feeding and production management of animals [35] but also to confirm their authenticity.

## 4. Materials and Methods

### 4.1. Materials

Samples of six different types of ripening cheeses (cheeses with internal mold (Blue, Gorgonzola)—HBC, cheeses with exterior mold (Camembert, Brie)—LBC, Swiss type semi-hard cheeses (Emmentaler)—SUI, Italian type very hard cheeses (Parmesan)—ITA, English type hard cheeses (Cheddar)—ENG and Dutch type semi-hard cheeses (Gouda, Edam)—NED), curd cheese (CC), and processed cheese (PC) were purchased in groceries in Warsaw. All of the investigated cheeses were made from cow’s milk. According to the manufacturer’s information on the package HBC and LBC were prepared from raw milk whereas other types were prepared from pasteurized milk. For PC the cheese of origin were NED, according to the manufacturer’s information on the package. Twelve different brands of each type of cheese with different place of origin were analyzed (*n* = 8 types × 12 brands = 96 samples). Three parallel samples of each different brand were purchased (*n* = 96 × 3 = 288 analytical samples). They were stored in original packaging at 4 °C in dark. All analyses were performed from fresh-opened cheese sample in triplicates (giving the exact number of analyses 288 × 8 = 864).

### 4.2. Fat Content Determination

Fat content in examined cheese samples was determined gravimetrically after tree-times extraction with a mixture of chloroform/methanol (*v*/*v* 2:1, 3 × 4.5 mL, room temperature), filtration through filter paper (84 g/m^2^; wash 4.5 mL of the mixture) and solvents evaporation under a stream of nitrogen, according to the procedure described by Folch et al. [46]. Three parallel samples were performed for each fresh-opened cheese.

### 4.3. Fatty Acids Analysis by GC-MS

FA profiles of examined cheeses were determined as fatty acid methyl esters (FAME) by capillary gas chromatography coupled with mass spectrometry (GC-MS). Prior to chromatographic analyses, FA were derivatized by the base- and acid-catalyzed methylation procedures [47] and then quantified using gas chromatograph coupled with mass spectrometer (Shimadzu GC-MS-QP2010 Plus EI, Tokyo, Japan); chromatographic system was equipped with a BPX70 fused silica column (120 m × 0.25 mm i.d. × 0.25 μm film thickness; Phenomenex, Torrance, CA, USA), a quadruple mass selective detector (Model 5973N, Shimadzu, Tokyo, Japan) and an injection port [48]. Nonadecanoic acid (99%, Sigma, St. Louis, MO, USA) was used as the internal standard (IS). FAME identification was based on electron impact ionization spectra of FAME and compared to authentic FAME standards (Supelco 37 Component FAME Mix, Sigma, St. Louis, MO, USA and Bacterial Acid Methyl Ester (BAME) Mix, Sigma, St. Louis, MO, USA), c9,t11C18:2—RA methyl ester standard (Sigma, St. Louis, MO, USA), t10,c12C18:2 (Sigma, St. Louis, MO, USA), c9t11c13C18:3—PA methyl ester standard (Methyl punicate, Matreya LCC, State College, PA, USA), c9t11t13C18:3—α-eleostearic ESA alpha methyl ester standard (Larodan Fine Chemicals, Solna, Sweden) and the NIST 2007 reference mass spectra library (National Institute of Standard and Technology, Gaithersburg, MD, USA). All FAME analyses were based on total ion current chromatograms and/or selected-ion monitoring chromatograms. Three parallel samples were prepared from each cheese sample. Results are expressed as μg/g of cheese.

### 4.4. Determination of CFA Content Using Ag^+^-HPLC-DAD

CFA in examined samples of cheese were analyzed as free FA, without any derivatization, with argentometric high performance liquid chromatography with photodiode array detection (Ag^+^-HPLC-DAD). Prior to chromatographic analyses samples of cheese were subjected to alkaline hydrolysis according to the method of Czauderna et al. [47]. Briefly, 50–100 mg of cheese samples was treated with a mixture of KOH in water and KOH in methanol solutions. Next, 25 μL of sorbic acid (Sigma, St. Louis, MO, USA) solution (0.4 mg/mL of chloroform), as the IS, was added. The mixture was flushed with argon (Ar), vortexed and sonicated under a stream of Ar at 95 °C for 10 min. The obtained mixture was protected from the light and stored in the sealed vial at ~22 °C overnight. Next, water was added to the hydrolysate and the solution was acidified with 4 M HCl to ~pH 2. Free FA were then extracted with methylene chloride (2 × 1.5 mL) and n-hexane (2 × 1.5 mL). The combined organic layers were dehydrated with anhydrous Na_2_SO_4_, filtered and removed under the stream of Ar. The obtained residue was dissolved in 0.5 mL of *n*-hexane, vigorously vortexed and transferred into the vial and then 5 μL of the resulting solution was injected on to the HPLC system. A Waters HPLC 625LC system (Waters, Milford, MA, USA) equipped with a photodiode array detector (DAD) (Waters, Milford, MA, USA) operated in a UV range from 195 to 400 nm was used for detection of CFA isomers. Four analytical ion-exchange columns loaded with silver ions (Chrompack ChromSpher, 5 μm, Lipids, 250 mm × 4.6 mm; Varian, Middelburg, the Netherlands) were used in conjunction with a guard column of 10 × 3 mm containing the same stationary phase. The ambient temperature was 22–24 °C, while a column heater maintained the temperature at 23 °C. Ag^+^-HPLC-DAD system pressure was 15.25 ± 0.08 MPa. The samples were subjected to isocratic elution (2 mL/min) using a mobile phase composed of *n*-hexane, glacial acetic acid and acetonitrile (98.4:1.6:0.0125, *v*/*v*/*v*). The columns were equilibrated with freshly prepared mobile phase at least 35 min before sample injection. The mobile phase was carefully stirred before chromatographic analysis as the reproducibility of the fractionation was sensitive to small fluctuations in the concentration of acetic acid and more to the concentration of acetonitrile. Detection of CFA was conducted at 234 nm for FA containing two conjugated double bonds (conjugated dienes—CD) and at 270 nm for FA with three conjugated double bonds (conjugated trienes—CT). IS was monitored at 259 nm. Identification of CFA as CD or CT was based on retention times and UV spectra of analytical standards of CLA: c9,t11C18:2; t10,c12C18:2; and CLA isomer mixture and CLnA: t8t10c12C18:3—α-calendic; t9t11c13C18:3—catalpic (CA); c9t11t13C18:3—α-eleostearic (ESA alpha); t9t11t13C18:3—β-eleostearic (ESA beta); and c9t11c13C18:3—punicic acid (PA) (Larodan Fine Chemicals, Solna, Sweden). Results are expressed as µg/g of cheese.

### 4.5. Statistical Analysis

All data were presented as mean values ± standard deviation. For variables with skew distribution, data were transformed into logarithms, retransformed after calculations and presented as mean and confidence interval. Statistica 12 software (StatSoft, Cracow, Poland) [49] was used for the statistical analysis. All variables were tested with one-way ANOVA and post-hoc HSD Tukey test. The acceptable level of significance was established at *p* ≤ 0.05.

### 4.6. Chemometric Analyses

In order to better understand the data trends, chemometric procedures were applied, where FA and CFA were used as chemical descriptors to study a possible discrimination of the cheese samples. Statistica 12 software (StatSoft, Cracow, Poland) [49] was used for the statistical analysis. Prior to analyses, the original data were transformed into natural logarithms and then standardized. Cluster analysis (CA) was performed to determine the similarity of the samples described by the set of variables. This analysis was carried out using the agglomeration method. Moreover, the Euclidean distance was used as the distance determination method and the Ward method was used as the agglomeration method. Next, in order to provide a first evaluation of discriminating efficiency of the FA and CFA composition, principal component analysis (PCA) was performed with standardized Varimax rotation. The factors were distinguished based on eigenvalues greater than 1. The calculated factor values were compared between the groups using the Kruskal-Wallis test with the assumed significance level *p* ≤ 0.01. Finally, in order to obtain appropriate classification rules for the cheese samples, a linear discriminant analysis (LDA) was performed. In order to optimize LDA, t Relevant discriminant functions were calculated in a stepwise progressive method, with the adopted tolerance value 1 − R^2^ = 0.01.

## 5. Conclusions

Results of present study clearly demonstrated that FA and CFA content may not only to contribute to the overall nutritional characteristics of cheese but also may be used as chemical biomarkers for assessing the origin and/or the type of ripening cheeses, which is of utmost importance for consumers. PCA of the FA and CFA content data not only enabled the differentiation of ripening cheeses from fresh cheeses and processed cheeses but also allowed to differentiate some types of ripening cheese. Proposed LDA model (based on 22 analyzed variables, mainly FA content) enabled assessing cheeses type with average predictive sensitivities of 86.5%. Therefore FA and CFA profiles coupled with chemometric techniques can be used as confirmation of cheeses’ authenticity.

## Figures and Tables

**Table 1:** Content of saturated fatty acids (SFA) in examined types of cheese (µg/g of cheese).

	CC	PC	NED	SUI	LBC	ITA	HBC	ENG	*p*-Value
C6:0	346 ± 107 ^a,b,c,d,e,f^	889 ± 490 ^g,h,i,j,k^	2326 ± 822 ^a,g^	2644 ± 962 ^b,h^	1712 ± 530 ^c,l^	3069 ± 904 ^d,i,l,m^	2164 ± 1032 ^e,j^	1958 ± 827 ^f,k,m^	<0.0001
C8:0	277 ± 60 ^a,b,c,d,e,f^	686 ± 318 ^g,h,i,j,k^	1677 ± 637 ^a,g^	2156 ± 684 ^b,h,l,m^	1251 ± 360 ^c,l,n^	2285 ± 593 ^d,i,n,o^	1844 ± 630 ^e,j^	1423 ± 516 ^f,k,m,o^	<0.0001
C9:0	23.3 ± 9.6 ^a,b,c^	41.9 ± 18.5 ^d^	99.6 ± 61.4 ^a,e^	<0.01 ^e,f,g,h,i^	55.4 ± 12.3 ^f,j^	130 ±45.3 ^b,d,g,j,k,l^	69.1 ± 31.1 ^c,h,k^	65.4 ± 49.0 ^i,l^	<0.0001
C10:0	646 ± 130 ^a,b,c,d,e,f^	1777 ± 893 ^g,h,i,j,k^	4511 ± 1890 ^a,g^	5803 ± 2063 ^b,h,l,m^	3155 ± 851 ^c,l,n,o^	5892 ± 1552 ^d,i,n,p^	5112 ± 1540 ^e,j,o^	3910 ± 1537 ^f,k,m,p^	<0.0001
C11:0	<0.01	34.3 (25.3–46.5) *	96.9 ± 94.8	86.5 (54.2–138) *	47.3 ± 18.4	141 (67.6–294) *	97.3 ± 39.8	64.3 ± 40.0	n.s.
C12:0	762 ± 148 ^a,b,c,d,e,f^	2208 ± 1001 ^g,h,i,j,k^	5372 ± 2201 ^a,g^	7107 ± 2510 ^b,h,l^	3457 ± 1460 ^c,l,m,n^	7237 ± 1939 ^d,i,m^	6399 ± 1950 ^e,j,n^	5141 ± 2108 ^f,k^	<0.0001
i-C13:0	9.30 ± 6.84 ^a,b,c^	33.6 ± 21.2 ^d,e^	83.5 ± 31.8 ^a,d,f^	96.1 ± 34.7 ^b,e,g,h,i^	41.8 ± 15.1 ^f,g^	81.7 ± 39.4 ^c^	52.6 ± 29.5 ^h^	46.5 ± 27.8 ^i^	<0.0001
C13:0	19.9 ± 10.4 ^a,b,c,d,e,f^	52.9 ± 34.9 ^g,h,i^	117 ± 51 ^a,j,k^	200 ± 94 ^b,g,j,l,m^	94.2 ± 38.2 ^c,l,n,o^	241 ± 68 ^d,h,k,n,p^	180 ± 59 ^e,i,o^	116 ± 67 ^f,m,p^	<0.0001
i-C14:0	27.0 ± 15.5	57.2 ± 44.4	201 ± 62	266 ± 113	125 ± 47	226 ± 108	175 ± 82	140 (58.0-338) *	n.s.
C14:0	3040 ± 551 ^a,b,c,d,e,f^	8042 ± 3937 ^g,h,i,j,k^	20255 ± 7526 ^a,g^	25501 ± 8005 ^b,h,l^	13868 ± 5938 ^c,l,m,n^	26208 ± 6900 ^d,i,m,o^	23267 ± 6753 ^e,j,n^	17567 ± 7770 ^f,k,o^	<0.0001
i-C15:0	46.7 ± 15.2 ^a,b,c,d,e,f^	122 ± 60 ^g,h,i,j,k^	292 ± 97 ^a,g^	374 ± 144 ^b,h,l^	212 ± 98 ^c,l,m^	363 ± 94 ^d,i,m^	300 ± 87 ^e,j^	259 ± 107 ^f,k^	<0.0001
a-C15:0	117 ± 23 ^a,b,c,d,e,f^	261 ± 129 ^g,h,i,j,k,l^	648 ± 210 ^a,g^	787 ± 236 ^b,h,m^	520 ± 153 ^c,j,m,n^	791 ± 258 ^d,j,n^	721 ± 232 ^e,k^	595 ± 230 ^f,l^	<0.0001
C15:0	306 ± 53 ^a,b,c,d,e,f^	735 ± 343 ^g,h,i,j,k^	1622 ± 592 ^a,g^	2046 ± 692 ^b,h^	1396 ± 411 ^c,l^	2262 ± 634 ^d,i,l,m^	1999 ± 581 ^e,j^	1585 ± 609 ^f,k,m^	<0.0001
i-C16:0	83.2 ± 20.7 ^a,b,c,d,e,f^	231 ± 122 ^g,h,i,j^	461 ± 196 ^a,g^	631 ± 220 ^b,h,k,l^	414 ± 136 ^c,k^	542 ± 166 ^d,i,m^	525 ± 143 ^e,j,n^	315 ± 115 ^f,l,m,n^	<0.0001
C16:0	9616 ± 1432 ^a,b,c,d,e,f^	23989 ± 9877 ^g,h,i,j,k,l^	51263 ± 16786 ^a,g^	62267 ± 17332 ^b,h^	44064 ± 12815 ^c,i,m^	67458 ± 16150 ^d,j,m,n^	59025 ± 15766 ^e,k^	44676 ± 19560 ^f,l,n^	<0.0001
i-C17:0	79.2 ± 13.6 ^a,b,c,d,e,f^	158 ± 84 ^g,h,i^	358 ± 103 ^a^	435 ± 171 ^b,g^	344 ± 106 ^c^	467 ± 119 ^d,h^	567 ± 479 ^e,i^	373 ± 115 ^f^	<0.0001
a-C16:0	188 ± 37 ^a,b,c,d,e,f^	424 ± 190 ^g,h,i,j,k,l^	968 ± 355 ^a,g^	1196 ± 433 ^b,h^	862 ± 251 ^c,i,m^	1276 ± 341 ^d,j,m^	1135 ± 318 ^e,k^	934 ± 306 ^f,l^	<0.0001
2,6,10,14-metylC15:0	22.0 (7.8–62.1) *^,a^	22.1 ± 20.0 ^b^	77.6 ± 62.9	108 (84.1–139) *^c^	70.3 ± 31.7	75.0 (49.7–113) *^,d^	84.6 (54.20–132) *^,e^	116 ± 63 ^a,b,c,d,e^	<0.0001
3,7,11,15-metylC16:0	12.5 (5.8–26.9) *^,a^	19.2 (10.8-33.4) *^,b^	54.4 (39.8–74.2) *	<0.01 ^c^	36.9 (25.4–53.6) *	<0.01 ^d^	53.8 (24.3–119) *	102 (59.3–174) *^,a,b,c,d^	0.0005
C17:0	175 ± 69 ^a,b,c,d,e,f^	427 ± 190 ^g,h,i,j,k,l^	928 ± 256 ^a,g^	990 ± 378 ^b,h^	895 ± 278 ^c,i^	1061 ± 317 ^d,j^	1022 ± 228 ^e,k^	814 ± 234 ^f,l^	<0.0001
i-C18:0	16.2 (13.3–19.7) *^,a,b^	18.6 (7.3–47.1) *^,c,d^	41.8 ± 36.0	125 (79.9–196) *	61.0 ± 21.2 ^a,c^	76.3 (31.9–182) *	59.6 ± 28.1 ^b,d^	42.0 (19.5–90.5) *	0.0013
C18:0	3003 ± 567 ^a,b,c,d,e,f^	6677 ± 3039 ^g,h,i,j,k,l^	14317 ± 4281 ^a,g^	15115 ± 3406 ^b,h^	14506 ± 5072 ^c,i^	16210 ± 4337 ^d,j^	16652 ± 4405 ^e,k^	14330 ± 4261 ^f,l^	<0.0001
C20:0	34.2 ± 7.2 ^a,b,c,d,e,f^	75.8 ± 38.5 ^g^	141 ± 58 ^a,g^	125 ± 64 ^b^	116 ± 45 ^c^	125 ± 73 ^d^	136 ± 46 ^e^	115 ± 44 ^f^	<0.0001
C22:0	3.4 ± 10.7 ^a,b^	<0.01 ^c,d^	17.8 (4.4–71.2) *^,e^	<0.01 ^f,g^	31.8 ± 20.0 ^a,c,e,f,h,i^	<0.01 ^h,j^	2.4 ± 8.2 ^i,k^	30.8 (18.1–52.3) *^,b,d,g,j,k^	<0.0001
C24:0	<0.01 ^a^	<0.01 ^b^	<0.01 ^c^	<0.01 ^d^	<0.01 ^e^	<0.01 ^f^	<0.01^g^	14.4 (10.5–19.8) *^,a,b,c,d,e,f,g^	<0.0001
SFA	15824 ± 2567 ^a,b,c,d,e,f^	40207 ± 17543 ^g,h,i,j,k,l^	91466 ± 31634 ^a,g^	112102 ± 33155 ^b,h,m^	74741 ± 21852 ^c,i,m,n^	119823 ± 29614 ^d,j,n,o^	104942 ± 28714 ^e,k^	80394 ± 33805 ^f,l,o^	<0.0001

Data are presented as mean values ± standard deviation. Variables with skew distribution were transformed into logarithms, retransformed after calculations and presented as mean and confidence interval (*). Values in a row sharing the same letter (a–p) are statistically different. *p*-Value ≤ 0.05 was considered significant. CC—Curd cheese, PC—Processed cheese, NED—Dutch type cheese, SUI—Swiss type cheese, LBC—Lichen blue cheese, ITA—Italian type cheese, HBC—Hypertrophied blue cheese, ENG—English type cheese, SFA—Saturated fatty acids, i—*iso-,* a—*anteiso-.*

**Table 2:** Content of MUFA in examined types of cheese (µg/g of cheese).

	CC	PC	NED	SUI	LBC	ITA	HBC	ENG	*p*-Value
C10:1	61.1 ± 21.9 ^a,b,c,d,e,f^	180 ± 112 ^g,h,i,j,k^	426 ± 138 ^a,g^	520 ± 196 ^b,h,l^	312 ± 89 ^c,l,m^	571 ± 201 ^d,i,m^	451 ± 144 ^e,j^	390 ± 202 ^f,k^	<0.0001
c9C12:1	16.5 ± 15.6 ^a,b,c,d,e^	51.8 ± 47.7 ^f,g^	136 ± 77 ^a^	189 ± 129 ^b,f,h^	78 ± 39 ^h,i^	210 ± 97 ^c,g,i^	124 ± 39 ^d^	122 ± 85 ^e^	<0.0001
c11C12:1	18.4 (7.14–47.5) *^,a,b,c,d,e^	27.9 (11.8–66.3) *^,f,g,h,i,j^	155 ± 102 ^a,f^	187 ± 80 ^b,g,k^	83.1 ± 32.1 ^k^	161 ± 126 ^c,h^	133 ± 39 ^d,i^	124 ± 65 ^e,j^	<0.0001
c9c14:1	247 ± 50 ^a,b,c,d,e,f^	603 ± 277 ^g,h,i,j,k^	1538 ± 592 ^a,g^	2060 ± 633 ^b,h,l^	1216 ± 347 ^c,l,m^	2103 ± 643 ^d,i,m^	1800 ± 588 ^e,j^	1501 ± 808 ^f,k^	<0.0001
t9C16:1	16.2 ± 13.5 ^a,b,c^	64.7 (36.5–114) *^,d,e^	131 ± 71.1 ^a,d,f^	125 (51.0–304) *	79.1 ± 33.1	70.5 (49.0–101) *^,f,g^	106 ± 53 ^b^	128 ± 72 ^c,e,g^	<0.0001
c7C16:1	50.7 ± 17.6 ^a,b,c,d,e,f^	123 ± 58 ^g,h,i,j,k^	286 ± 99 ^a,g^	382 ± 132 ^b,h,l^	240 ± 66 ^c,l^	304 ± 129 ^d,i^	290 ± 93 ^e,j^	277 ± 123 ^f,k^	<0.0001
c9C16:1	418 ± 129 ^a,b,c,d,e,f^	1079 ± 521 ^g,h,i,j,k,l^	2559 ± 910 ^a,g^	3110 ± 891 ^b,h^	2124 ± 631 ^c,i,m^	3277 ± 912 ^d,j,m^	2855 ± 1232 ^e,k^	2365 ± 871 ^f,l^	<0.0001
t7C17:1	<0.01 ^a^	<0.01 ^b^	<0.01 ^c^	<0.01 ^d^	<0.01 ^e^	<0.01 ^f^	<0.01 ^g^	12.0 ± 5.2 ^a,b,c,d,e,f,g^	0.0185
t9C17:1	<0.01 ^a,b^	<0.01 ^c^	66.4 (35.5–124) *^,a,d,e,f^	<0.01 ^d,g^	<0.01 ^e,h^	<0.01 ^f,i^	75.5 (37.7–151) *	52.3 ± 48.9 ^b,c,g,h,i^	<0.0001
c9C17:1	59.6 ± 20.3 ^a,b,c,d,e,f^	130 ± 62 ^g,h,i,j,k^	317 ± 141 ^a,g^	349 ± 139 ^b,h^	232 ± 72 ^c^	297 ± 95 ^d,i^	329 ± 84 ^e,j^	275 ± 119 ^f,k^	<0.0001
t6C18:1	<0.01	27.3 ± 1.4	37.2 (9.15–151) *	<0.01	85.6 (13.5–541) *	142 (68.6–294) *	71.6 ± 44.1	40.4 ± 30.1	n.s.
t8C18:1	32.5 ± 14.8 ^a,b,c,d,e,f^	66.2 ± 50.7 ^g,h,i^	154 ± 79 ^a^	219 ± 98 ^b,g,j^	153 ± 54 ^c^	199 ± 91 ^d,h^	213 ± 91 ^e,i,k^	124 ± 28 ^f,j,k^	<0.0001
t9C18:1	35.0 ± 9.6 ^a,b,c,d,e,f^	98.4 ± 48.4 ^g,h,i,j^	213 ± 83 ^a,g^	219 ± 70 ^b,h^	201 ± 71 ^c^	210 ± 121 ^d,i^	290 ± 126 ^e,j^	190 ± 63 ^f^	<0.0001
t11C18:1	63.5 ± 19.1 ^a,b,c,d,e,f^	138 ± 71 ^g,h,i^	347 ± 183 ^a,j^	490 ± 162 ^b,g^	343 ± 113 ^c,k^	534 ± 311 ^d,h^	613 ± 279 ^e,i,j,k,l^	310 ± 89 ^f,l^	<0.0001
c6C18:1	267 ± 123 ^a,b,c,d,e,f^	488 ± 364 ^g,h,i,j,k,l^	1387 ± 617 ^a,g^	1417 ± 469 ^b,h^	1249 ± 475 ^c,i^	1221 ± 477 ^d,j,m^	1387 ± 561 ^e,k^	1873 ± 767 ^f,l,m^	<0.0001
c7C18:1	50.8 ± 10.1 ^a,b,c,d,e,f,g^	224 ± 252 ^a,h,i^	244 ± 104 ^b,j,k^	353 ± 77^c^	282 ± 86 ^d,l^	460 ± 139 ^e,h,j,l,m^	431 ± 188 ^f,i,k,n^	258 ± 60 ^g,m,n^	<0.0001
c9C18:1	6292 ± 1097 ^a,b,c,d,e,f^	15218 ± 6234 ^g,h,i,j,k,l^	35149 ± 11413 ^a,g^	40203 ± 10429 ^b,h^	29574 ± 9286 ^c,i^	40999 ± 10918 ^d,j^	38925 ± 10440 ^e,k^	29247 ± 11146 ^f,l^	<0.0001
c10C18:1	40.7 ± 11.4 ^a,b,c,d,e,f^	86.4 ± 61.4 ^g,h,i,j,k,l^	220 ± 114 ^a,g^	230 ± 87 ^b,h^	221 ± 76 ^c,i^	298 ± 91 ^d,j^	323 ± 129 ^e,k^	266 ± 86 ^f,l^	<0.0001
c11C18:1	121 ± 24 ^a,b,c,d,e,f^	241 ± 126 ^g,h,i,j,k^	570 ± 246 ^a,g,l^	755 ± 243 ^b,h,m^	589 ± 186 ^c,i,n^	771 ± 213 ^d,j,o^	916 ± 340 ^e,k,l,n,p^	477 ± 126 ^f,m,o,p^	<0.0001
c12C18:1	35.2 ± 9.1 ^a,b,c,d,e^	148 ± 103 ^f,g^	233 ± 86 ^a,h^	274 ± 118 ^b,i^	208 ± 72 ^c,j,k^	411 ± 185 ^d,f,h,j,l^	364 ± 178 ^e,g,k,m^	125 ± 51 ^i,l,m^	<0.0001
c13C18:1	54.4 ± 15.7 ^a,b,c,d,e,f^	141 ± 106 ^g,h,i,j,k,l^	311 ± 106 ^a,g^	361 ± 107 ^b,h^	287 ± 97 ^c,i^	339 ± 108 ^d,j^	405 ± 134 ^e,k^	349 ± 113 ^f,l^	<0.0001
c14C18:1	31.9 ± 12.0 ^a,b,c,d,e,f^	85.6 ± 58.2 ^g,h,i,j^	160 ± 63 ^a^	206 ± 114 ^b,g^	169 ± 55 ^c,h^	219 ± 65 ^d,i,k^	219 ± 53 ^e,j,l^	136 ± 56 ^f,k,l^	<0.0001
c15C18:1	6.38 ± 12.3	<0.01 ^a^	38.6 ± 10.9	18.3 ± 13.5	<0.01 ^b^	<0.01 ^c^	41.9 ± 25.6 ^a,b,c^	35.8 ± 11.6	0.0008
c9C20:1	29.7 ± 11.9 ^a,b,c,d,e^	75.0 ± 52.7	151 ± 93 ^a^	115 ± 93 ^b^	120 ± 37 ^c^	150 ± 72 ^d^	147 ± 54 ^e^	105 ± 70	0.0001
c11C20:1	20.1 ± 13.6 ^a,b^	24.6 ± 23.0	99.6 ± 91.2 ^a^	83.5 ± 18.2	49.8 ± 21.2	103 ± 35.0 ^b^	84.3 ± 34.3	46.9 ± 29.2	0.0027
MUFA	7971 ± 1390 ^a,b,c,d,e,f^	19221 ± 8058 ^g,h,i,j,k,l^	44786 ± 14481 ^a,g^	51980 ± 13385 ^b,h^	40329 ± 16561 ^c,i^	52608 ± 14087 ^d,j^	50464 ± 13899 ^e,k^	38879 ± 14103 ^f,l^	<0.0001

Data are presented as mean values ± standard deviation. Variables with skew distribution were transformed into logarithms, retransformed after calculations and presented as mean and confidence interval (*). Values in a row sharing the same letter (a–p) are statistically different. *p*-Value ≤ 0.05 was considered significant. CC—Curd cheese, PC—Processed cheese, NED—Dutch type cheese, SUI—Swiss type cheese, LBC—Lichen blue cheese, ITA—Italian type cheese, HBC—Hypertrophied blue cheese, ENG—English type cheese, MUFA—Monounsaturated fatty acids, c—*cis,* t—*trans*.

**Table 3:** Content of PUFA in examined types of cheese (µg/g of cheese).

	CC	PC	NED	SUI	LBC	ITA	HBC	ENG	*p*-Value
t9t12C18:2	25.0 ± 9.1 ^a,b,c,d,e^	60.0 ± 32.3 ^f^	143 ± 5 ^a^	155 ± 113 ^b^	126 ± 43	167 ± 81 ^c^	149 ± 57 ^d^	200 ± 191 ^e,f^	0.0001
t9c12C18:2	45.8 ± 22.7 ^a,b,c,d,e,f^	129 ± 77 ^g,h,i,j,k^	296 ± 152 ^a,g^	351 ± 114 ^b,h^	236 ± 74 ^c^	320 ± 115 ^d,i^	402 ± 169 ^e,j^	331 ± 124 ^f,k^	<0.0001
t11c15C18:2	32.3 (18.8–554) *^,a,b,c,d,e,f^	57.7 (25.5–131) *^,g,h,i^	123 ± 68 ^a,g^	106 ± 81 ^b^	86 ± 30 ^c^	125 ± 76 ^d,h^	131 ± 54 ^e,i^	82.2 ± 35.1 ^f^	<0.0001
c9c12C18:2	715 ± 609 ^a,b,c^	1071 (466–2460) *^,d,e^	2334 ± 866 ^f,g^	3268 ± 2043 ^a^	2298 ± 93 ^h,i^	4898 ± 1992 ^b,d,f,h,j^	4254 ± 1946 ^c,e,g,i,k^	1965 ± 601 ^j,k^	<0.0001
c6c9c12C18:3	40.6 (10.5–157) *^,a^	<0.01 ^b^	42.8 (23.6–77.7) *^c^	<0.01 ^d^	63.5 (56.0–72.1) *^,e^	<0.01 ^f^	59.8 ± 51.3 ^a,b,c,d,e,f,g^	23.9 (16.3–35.10) *^,g^	<0.0001
c9c12c15C18:3	104 ± 59 ^a,b,c,d,e,f^	211 ± 103 ^g,h,i,j,k^	538 ± 155 ^a,g^	695 ± 410 ^b,h^	411 ± 153 ^c,l^	713 ± 312 ^d,i,l^	628 ± 257 ^e,j^	540 ± 226 ^f,k^	<0.0001
c9t11C18:2	74.1 ± 40.7 ^a,b,c,d,e,f^	180 ± 108 ^g,h,i,j^	460 ± 166 ^a,g^	538 ± 178 ^b,h^	382 ± 140 ^c,k^	375 ± 235 ^d,l^	481 ± 166 ^e,i^	618 ± 281 ^f,j,k,l^	<0.0001
ttCLA	21.8 ± 16.6 ^a^	11.6 ± 10.5 ^b^	74.7 ± 31.9	<0.01 ^c,d^	45.6 ± 18.7 ^e^	149 ± 43.4 ^a,b,c,e,f^	81.5 ± 71.3 ^d^	37.8 ± 43.6 ^f^	<0.0001
c8c11c14C20:3	<0.01 ^a,b,c^	<0.01 ^d,e,f^	47.9 ± 47.8	<0.01 ^g^	59.8 ± 26.0 ^a,d^	131 (76.9–222) *^,b,e^	87.0 ± 38.2 ^c,f,g^	49.8 ± 39.3	<0.0001
c5c8c11c14C20:4	19.3 (13.0–28.7) *^,a,b,c^	41.3 (25.0–68.2) *^,d,e,f^	111 ± 77.7 ^a,d,g^	198 (144–271) *	51.0 ± 0.7 ^g,h,i^	111 ± 109 ^b,e,h^	131 ± 44.2 ^c,f,i^	90.1 ± 56.7	<0.0001
C18:3 conj1	-	-	-	-	-	-	-	31.7 (19.7–50.9) *	-
C18:3 conj2	-	-	-	-	-	-	<0.01	46.3 (11.3–190) *	-
c9t11c13C18:3	-	-	-	-	-	-	124 ± 50.9	36.2 ± 14	-
c4c7c10c13c16c19C22:6	4.5 ± 11.0 ^a^	<0.01 ^b,c^	65.0 ± 60.1 ^a,b,d,e,f^	<0.01^d^	<0.01 ^e^	<0.01 ^f,g^	62.9 (43.7–90.4) *	49.7 ± 49.4 ^c,g^	<0.0001
PUFA	1006 ± 673 ^a,b,c,d,e,f^	2128 ± 1690 ^g,h,i^	4145 ± 1310 ^a,j^	5363 ± 3203 ^b,g^	3677 ± 1316 ^c,k,l^	6818 ± 2638 ^d,h,j,k,m^	6434 ± 2451 ^e,i,l^	4146 ± 1379 ^f,m^	<0.0001
n3PUFA	115 ± 72 ^a,b,c,d,e,f^	262 ± 141 ^g,h,i,j,k^	726 ± 230 ^a,g^	801 ± 444 ^b,h^	497 ± 174 ^c^	838 ± 358 ^d,i^	784 ± 297 ^e,j^	679 ± 289 ^f,k^	<0.0001
n6PUFA	813 ± 656 ^a,b,c,d^	1685 ± 1596 ^e,f,g^	2941 ± 1082 ^a,h,i^	3858 ± 2169 ^b,e^	2757 ± 1043 ^j,k^	5556 ± 2162 ^c,f,h,j,l^	5083 ± 2184 ^d,g,i,k,m^	2651 ± 881 ^l,m^	<0.0001
n3/n6	0.18 ± 0.10	0.20 ± 0.08	0.26 ± 0.07 ^a^	0.21 ± 0.07	0.19 ± 0.04	0.16 ± 0.06 ^a,b^	0.17 ± 0.07 ^c^	0.26 ± 0.08 ^b,c^	0.0021
Sum FA	27838 ± 4403 ^a,b,c,d,e,f^	68295 ± 29842 ^g,h,i,j,k,l^	154921 ± 51350 ^a,g^	184739 ± 52291 ^b,h^	133527 ± 43339 ^c,i,m^	195781 ± 49692 ^d,j,m,n^	178723 ± 48910 ^e,k^	137825 ± 52496 ^f,l,n^	<0.0001

Data are presented as mean values ± standard deviation. Variables with skew distribution were transformed into logarithms, retransformed after calculations and presented as mean and confidence interval (*). Values in a row sharing the same letter (a–m) are statistically different. *p*-Value ≤ 0.05 was considered significant. CC—Curd cheese, PC—Processed cheese, NED—Dutch type cheese, SUI—Swiss type cheese, LBC—Lichen blue cheese, ITA—Italian type cheese, HBC—Hypertrophied blue cheese, ENG—English type cheese, PUFA—Polyunsaturated fatty acids, FA—Fatty acids, c—*cis,* t—*trans*.

**Table 4:** Conjugated fatty acids content in examined types of cheese (µg/g of cheese).

	CC	PC	NED	SUI	LBC	ITA	HBC	ENG	*p*-Value
CFA	399 ± 132 ^a,b,c,d,e,f^	700 ± 294 ^g,h,i,j,k^	1008 ± 268 ^a,l^	1248 ± 210 ^b,g,m^	1142 ± 229 ^c,h,n^	1138 ± 337 ^d,i,o^	1329 ± 383 ^e,j,p^	1736 ± 330 ^f,k,l,m,n,o,p^	<0.0001
CD	383 ± 133 ^a,b,c,d,e,f^	671 ± 309 ^g,h,i,j^	961 ± 259 ^a,k^	1058 ± 225 ^b,g,l^	1132 ± 233 ^c,h,m^	958 ± 270 ^d,n^	1237 ± 340 ^e,i^	1513 ± 342 ^f,j,k,l,m,n^	<0.0001
tt	62.0 ± 18.7 ^a,b,c,d,e^	94.9 ± 35.5 ^f^	80.5 ± 13.4 ^g,h,i,j^	117 ± 22 ^a,g,k^	116 ± 26 ^b,l^	127 ± 33 ^c,h,m^	124 ± 24 ^d,i,n^	171 ± 39 ^e,f,j,k,l,m,n^	<0.0001
ct	320 ± 115 ^a,b,c,d,e,f^	574 ± 287 ^g,h,i,j^	881 ± 247 ^a,k^	941 ± 205 ^b,g,l^	1013 ± 209 ^c,h,m^	828 ± 246 ^d,n^	1100 ± 329 ^e,i^	1342 ± 310 ^f,j,k,l,m,n^	<0.0001
cc	0.98 ± 0.37 ^a,b^	2.21 ± 1.19 ^c,d,e.f^	<0.01 ^c,g,h,i^	<0.01 ^d,j,k,l^	3.72 ± 1.07 ^a,g,j,m^	4.83 (2.80–8.32) *^,h,k,n,o^	4.30 ± 1.89 ^b,e,i,l,n,p^	<0.01 ^f,m,o,p^	<0.0001
c9t11CLA	281 ± 105 ^a,b,c,d,e,f^	507 ± 256 ^g,h,i^	779 ± 228 ^a,j^	828 ± 187 ^b^	879 ± 198 ^c,g^	729 ± 227 ^d,k^	978 ± 299 ^e,h^	1123 ± 446 ^f,i,j,k^	<0.0001
CT	16.1 ± 12.9 ^a,b,c^	11.1 (3.24–38.1) *^,d,e,f^	47.3 ± 46.9 ^g,h,i^	190 ± 68.6 ^a,d,g,j,k^	4.20 (1.16–15.2) *^,j,l,m,n^	180 ± 89 ^b,e,h,l,o^	40.5 (9.35–175) *^,k,m,o,p^	223 ± 68 ^c,f,i,n,p^	<0.0001
ttt	13.9 ± 13.0 ^a,b,c^	83.9 (44.0–160) *^,d,e,f^	27.4 (12.4–60.7) *^,g,h,i^	173 ± 65 ^a,d,g,j,k^	3.10 (0.94–10.2) *^,j,l,m^	172 ± 91 ^b,e,h,l,n^	28.4 (3.30–244) *^,k,n,o^	185 ± 72 ^c,f,i,m,o^	<0.0001
ttc/ctt	0.82 ± 0.66 ^a,b^	2.14 ± 1.18 ^c,d^	4.17 ± 3.92 ^e^	12.4 ± 7.4 ^a,c,f,g^	1.49 (0.38–5.86) *^,f,h^	1.51 (0.37–6.13) *^,i^	3.36 (0.88–12.9) *^,j^	31.5 ± 13.2 ^b,d,e,g,h,i,j^	<0.0001
cct	1.36 ± 1.31	1.11 (0.24–5.24) *	1.44 (0.27–7.66) *	2.34 (0.73–7.48) *	0.54 (0.12–2.49) *	4.00 ± 3.08	3.76 (1.28–11.1) *	2.17 (0.28–17.0) *	n.s.

Data are presented as mean values ± standard deviation. Values in a row sharing the same letter (a–p) are statistically different (*p* < 0.05) in Tukey test. Variables with skew distribution were transformed into logarithms, retransformed after calculations and presented as mean and confidence interval (*). CC—Curd cheese, PC—Processed cheese, NED—Dutch type cheese, SUI—Swiss type cheese, LBC—Lichen blue cheese, ITA—Italian type cheese, HBC—Hypertrophied blue cheese, ENG—English type cheese, PUFA—Polyunsaturated fatty acids, FA—Fatty acids, c—*cis,* t—*trans*, CFA—Conjugated fatty acids, CD—Conjugated dienes, CLA—Conjugated linoleic acids, CT—Conjugated trienes, n.s.—Not significant.

**Table 5:** The comparison of experimental groups regarding identified factors.

	CC	PC	NED	SUI	LBC	ITA	HBC	ENG	*p*-Value
PC1	**12.67 ± 3.20 ^a,b,c,d,e,f^**	**5.20 ± 2.94 ^g,h,I,j^**	**−2.30 ± 1.78 ^a^**	**−3.59 ± 1.76 ^b,g^**	**−1.38 ± 2.09 ^c^**	**−4.26 ± 2.26 ^d,h^**	**−3.99 ± 1.71 ^e,i^**	**−2.33 ± 2.19 ^f,j^**	<0.0001
PC2	**0.06 ± 0.79 ^a^**	**1.44 ± 2.30 ^b,c,d^**	**0.11 ± 1.91 ^e^**	**3.27 ± 0.89 ^a,e,f,g,h^**	**−2.68 ± 0.54 ^b,f,i^**	**2.17 ± 0.87 ^i,j,k^**	**−1.85 ± 1.15 ^c,g,j^**	**−2.52 ± 1.85 ^d,h,k^**	<0.0001
PC3	−**0.80 ± 0.91 ^a^**	**1.12 ± 4.21 ^b^**	**−0.28 ± 0.41 ^c^**	**−0.67 ± 0.50 ^d^**	**1.03 ± 0.33 ^a,b,c,d,e^**	**−0.67 ± 0.51 ^e^**	0.16 ± 0.49	0.11 ± 0.65	<0.0001
PC4	−**0.02 ± 1.45 ^a^**	**0.18 ± 0.86 ^b^**	**−0.47 ± 0.43 ^c^**	**−0.85 ± 0.53 ^d,e,f^**	**−1.94 ± 0.97 ^c,d,g^**	**−0.83 ± 0.85 ^e,h^**	**−1.21 ± 0.94**	**2.86 ± 0.79**	<0.0001

Data is presented as mean ± SD. The boldfaced factors sharing the same letter showed significant differences in Dunn’s test (p < 0.05). CC—Curd cheese, PC—Processed cheese, NED—Dutch type cheese, SUI—Swiss type cheese, LBC—Lichen blue cheese, ITA—Italian type cheese, HBC—Hypertrophied blue cheese, ENG—English type cheese.

**Table 6:** Coefficients and average value of canonical variables included in the final model.

Coefficients of Canonical Variables			
Variable (Discriminatory Power)	DF1 (62.8%)	DF2 (15.8%)	DF3 (10.4%)
C14:0	−2.87486	−0.23823	6.15487
C15:0	0.41445	−0.88030	0.30208
C10:0	0.46920	−0.81715	−2.32960
C12:0	3.27553	0.21526	−2.48056
C16:0	−0.15933	−0.65019	−0.24584
c9C16:1	−0.53719	−0.62480	0.44081
c9C18:1	−0.38686	2.92710	−0.34280
a-C15:0	−1.59109	0.16220	−0.17732
c9C14:1	1.21217	−2.15447	−0.60549
C18:0	1.03007	−0.39936	−0.40455
i-C17:0	0.85398	−0.66452	0.54888
i-C15:0	0.03831	0.24962	0.11024
C6:0	−2.05800	0.10286	−0.58257
C8:0	−0.44120	0.35613	0.05495
c9C17:1	1.44316	−0.55230	−0.34508
C17:0	−0.58887	1.98339	0.71262
c13C18:1	−0.02018	−2.08316	−0.57036
c14C18:1	0.40469	−0.10663	0.81964
i-C18:0	−0.34923	0.20157	0.26644
c9C20:1	0.13310	0.12021	0.17548
t9t12C18:2	0.20479	0.11458	−0.33110
c9t11C18:2	0.11583	−0.81304	−0.78317
Average value of canonical variables			
CC	−6.75468	0.12994	0.17289
PC	−1.94973	0.96185	−1.03844
NED	1.03648	−0.20877	0.07775
SUI	2.15927	1.28350	−0.74175
LBC	0.34335	−1.28166	1.92010
ITA	2.23933	2.49492	0.80659
HBC	1.44598	−1.56363	0.86708
ENG	1.48000	−1.81616	−2.06422

CC—Curd cheese, PC—Processed cheese, NED—Dutch type cheese, SUI—Swiss type cheese, LBC—Lichen blue cheese, ITA—Italian type cheese, HBC—Hypertrophied blue cheese, ENG—English type cheese, c—*cis*, t—*trans*.

**Table 7:** Classification results of LDA presenting percentage predicted group membership for actual groups.

Actual Group	Correct Classification (%)	Predicted Group Membership							
		CC	PC	NED	SUI	LBC	ITA	HBC	ENG
CC	100	12	0	0	0	0	0	0	0
PC	83	0	10	1	0	0	1	0	0
NED	75	0	0	9	1	1	0	0	1
SUI	83	0	0	1	10	0	1	0	0
LBC	100	0	0	0	0	12	0	0	0
ITA	100	0	0	0	0	0	12	0	0
HBC	66	0	0	2	0	2	0	8	0
ENG	83	0	0	0	1	0	0	1	10
Σ	86	12	10	13	12	15	14	9	11

CC—Curd cheese, PC—Processed cheese, NED—Dutch type cheese, SUI—Swiss type cheese, LBC—Lichen blue cheese, ITA—Italian type cheese, HBC—Hypertrophied blue cheese, ENG—English type cheese.

## Supplementary Materials

The following are available online, Figure S1: Scree plot of principal components. Table S1: The matrix of factor analysis structure.
