# Supplementary material for: Chemometric Analysis of Fatty Acids Profile of Ripening Chesses
Source: Molecules. 2020 Apr 15;25(8):1814. doi: 10.3390/molecules25081814 (PMC7221737; doi:10.3390/molecules25081814)
Supplement: Supplementary file 1 [file molecules-25-01814-s001.pdf]

Chemometric Analysis of Fatty Acids Profile of Ripening Chesses

Agnieszka Białek [1,*], Małgorzata Białek [1,2,*], Tomasz Lepionka [3], Małgorzata Czerwonka [4] and Marian Czauderna [2]

1 Department of Animal Improvement and Nutrigenomics, Institute of Genetics and Animal Breeding, Polish Academy of Sciences, Postępu 36A Jastrzębiec, 05-552 Magdalenka, Poland
2 The Kielanowski Institute of Animal Physiology and Nutrition, Polish Academy of Sciences, Instytucka 3, 05-110 Jabłonna, Poland; mr.czauderna@gmail.com
3 Laboratory of Hygiene, Food and Nutrition, Military Institute of Hygiene and Epidemiology, Kozielska 4, 01-163 Warsaw, Poland; tomasz.lepionka@wihe.pl
4 Department of Bromatology, Medical University of Warsaw, Banacha 1, 02-097 Warsaw, Poland; malgorzata.czerwonka@wum.edu.pl
* Correspondence: a.bialek@ighz.pl (A.B.); m.bialek@ifzz.pl (M.B.); Tel.: +48-22-736-7128 (A.B.); +48-22-765-3350 (M.B.)

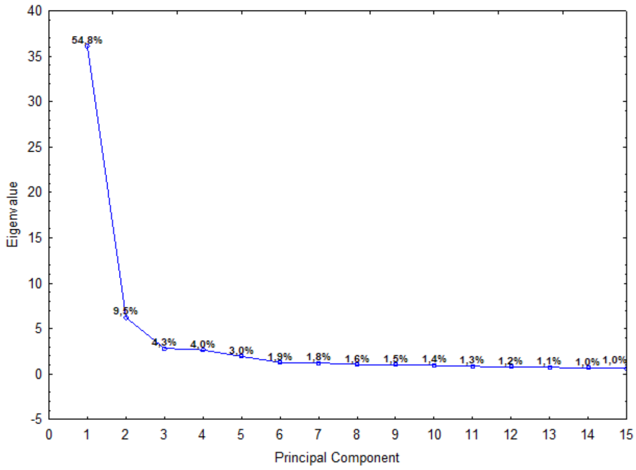

Figure S1. Scree plot of principal components.

Table S1. The matrix of factor analysis structure.

	PC1 54.8%	PC2 9.5%	PC3 4.3%	PC4 4.0%
CFA	**−0.8652**	−0.2092	0.0360	0.2682
CD	**−0.8376**	−0.3304	−0.0391	0.1939
c9t11CLA	**−0.6886**	−0.1855	0.1121	0.2590
C6:0	**−0.8389**	−0.3437	−0.0659	0.1828
C13:0	**−0.8180**	−0.3607	−0.0515	0.1893
i-C14:0	**−0.9135**	0.1848	0.0961	0.0372
C14:0	**−0.9069**	0.2164	0.0902	−0.0053
c9C14:1	**−0.9491**	0.1785	0.1243	−0.0228
a-C15:0	**−0.8757**	0.0452	−0.2985	−0.0703
C15:0	**−0.6063**	−0.4457	−0.0058	−0.1082
i-C16:0	**−0.9508**	0.1645	0.1249	−0.0071

C16:0	**−0.7967**	0.2410	0.0141	−0.0949
c7C16:1	**−0.7889**	0.2172	0.0090	−0.0171
c9C16:1	**−0.9518**	0.1646	0.1329	−0.0516
i-C17:0	**−0.9279**	0.1675	0.0833	0.0471
C17:0	**−0.9398**	0.1412	0.1436	−0.0089
c9C17:1	**−0.9452**	0.1074	0.0995	0.0386
C18:0	**−0.9544**	0.1051	0.1435	−0.0538
t9C18:1	**−0.8657**	0.1462	0.1060	−0.1638
t11C18:1	**−0.9437**	0.1523	0.1152	−0.1331
c6C18:1	**−0.8635**	0.1133	0.0570	−0.0593
c7C18:1	**−0.9474**	0.0933	0.1531	−0.0296
c9C18:1	**−0.9388**	0.0416	0.1196	−0.0165
c9c12C18:2	**−0.9109**	0.0076	0.1572	−0.1010
c9c12c15C18:3	**−0.9106**	0.0901	0.1267	−0.0167
c9t11C18:2	**−0.9460**	0.0112	0.1564	−0.0282
SFA	**−0.8467**	0.0457	0.1165	−0.1274
MUFA	**−0.8630**	0.0222	0.0563	−0.0936
PUFA	**−0.7937**	−0.2599	−0.4334	0.1361
n3PUFA	**−0.8990**	−0.0043	−0.1567	−0.1735
n6PUFA	**−0.9591**	0.0544	−0.0459	−0.0998
t6C18:1	**−0.8110**	0.1685	0.0041	−0.3166
c13C18:1	**−0.9182**	0.0416	0.1189	0.0600
c14C18:1	**−0.6605**	−0.2575	−0.1472	−0.0447
CT	**−0.8509**	0.0221	−0.2905	−0.2343
ttt	**−0.9152**	0.1119	0.0646	0.0577
ttc/ctt	**−0.7286**	−0.0931	−0.1197	−0.0806
cct	**−0.8975**	−0.0489	0.1578	0.1896
c6c9c12C18:3	**−0.9529**	0.1553	0.1197	−0.0450
c9C20:1	**−0.9506**	0.1651	0.1343	−0.0672
ttCLA	**−0.9553**	0.0256	−0.0686	−0.0641
c5c8c11c14C20:4	**−0.8747**	−0.0371	−0.3004	−0.1352
C22:0	**−0.9235**	0.0686	0.0982	0.0474
c4c7c10c13c16c19C22:6	**−0.8704**	−0.0132	−0.2954	−0.2013
C20:0	−0.1433	**−0.7744**	0.1066	−0.1340
Sum FA	−0.3646	**−0.7096**	0.2066	−0.1663
C11:0	−0.1260	**−0.7383**	0.1156	−0.0394
2,6,10,14-metylC15:0	−0.1888	**−0.6412**	0.1928	−0.1415
3,7,11,15-metylC16:0	−0.4377	**−0.6776**	0.1538	−0.1155
c8c11c14C20:3	−0.5112	−0.2945	**−0.7357**	−0.0530
tt	−0.4523	−0.2231	**−0.7531**	−0.1615
ct	−0.4685	0.3054	−0.1660	**0.6192**
C8:0	0.3465	−0.2850	0.2256	**−0.6343**

CFA—Conjugated fatty acids, CD—Conjugated dienes, CT—Conjugated trienes, c—*cis*, t—*trans*, i—*iso-*, a—*anteiso*, SFA—Saturated fatty acids, MUFA—Monounsaturated fatty acids, PUFA—Polyunsaturated fatty acids; the most significant loadings are boldfaced.
